# *Eubacterium limosum* modulates gut microbiota and produces anti-inflammatory metabolites to alleviate DSS-induced colitis

**DOI:** 10.3389/fimmu.2025.1728808

**Published:** 2025-12-16

**Authors:** Yao Lu, Huijing Tang, Qianhua Fan, Ruiting Lan, Xiaoying Lin, Shuwei Zhang, Liyun Liu, Jianguo Xu

**Affiliations:** 1 National Key Laboratory of Intelligent Tracking and Forecasting for Infectious Diseases, National Institute for Communicable Disease Control and Prevention, Chinese Center for Disease Control and Prevention, Beijing, China; 2 School of Biotechnology and Biomolecular Sciences, University of New South Wales, Sydney, NSW, Australia; 3 Department of Epidemiology, Center for Global Health, School of Public Health, Nanjing Medical University, Nanjing, Jiangsu, China; 4 Hebei Key Laboratory of Intractable Pathogens, Shijiazhuang Center for Disease Control and Prevention, Shijiazhuang, Hebei, China; 5 Research Center for Reverse Microbial Etiology, Workstation of Academician, Shanxi Medical University, Taiyuan, China

**Keywords:** *Eubacterium limosum*, gut microbiota, colitis, IL-17 signaling, metabolites

## Abstract

**Introduction:**

Inflammatory bowel disease (IBD) is a chronic inflammatory condition of the intestine, for which no cure currently exists. The gut microbiota play a critical role in ameliorating IBD, and *Eubacterium limosum* has emerged as a potential probiotic with anti-inflammatory properties. However, the specific anti-inflammatory effects of *E. limosum* and the underlying mechanisms remain largely unexplored.

**Methods:**

The *E. limosum* strain El1405 was utilized to evaluate its effects on dextran sodium sulfate (DSS)-induced murine colitis. The structural changes in intestinal microbiota were assessed using 16S rRNA gene sequencing. Inflammatory cytokines in the colon and serum were measured via enzyme-linked immunosorbent assay, while metabolomics was employed to analyze metabolites present in both the colon and serum.

**Results:**

The supplementation with *E. limosum* El1405 significantly reduced the disease activity index, colon shortening, and colonic histopathological lesions. El1405 reshaped the intestinal microbiota community structure, resulting in a significant increase in the abundance of *Bacteroides acidifaciens*, *Bacteroides thetaiotaomicron*, *Mucispirillum schaedleri, Phocaeicola vulgatus* (formerly *Bacteroides vulgatus*), and *Akkermansia muciniphila*, while concurrently decreasing the abundance of *Escherichia coli* and *Enterococcus faecalis*. The *E. limosum* intervention downregulated IL-17 signaling and reduced levels of inflammatory cytokines associated with IL-17 signaling, including IL-6, IL-17, TNF-α, IL-21, IL-22, and GM-CSF. *E. limosum* could induce anti-inflammatory effects by altering the serum metabolome of mice, especially producing anti-inflammatory metabolites such as indole-3-acetic acid and indole-3-lactic acid.

**Discussion:**

This study demonstrated the beneficial effects of *E. limosum* El1405 on DSS-induced colitis in mice, by modulating gut microbiota, reducing inflammatory cytokines in the colon and serum, and increasing anti-inflammatory metabolites in the serum. All contribute to the downregulation of IL-17 signaling and the alleviation of colitis. *E. limosum* supplementation may represent a promising probiotic candidate for IBD prevention.

## 1 Introduction

Inflammatory bowel disease (IBD) is a chronic inflammatory condition of the intestine, encompassing Crohn’s disease (CD) and ulcerative colitis (UC) (1). IBD is a disease with rising prevalence on all continents. The incidence and prevalence of IBD in developing countries are increasing annually (2). Over the past 20 years, numerous drugs have been developed and redesigned for the treatment of IBD. Current treatments for IBD primarily include 5-aminosalicylic acid compounds, immunosuppressants such as TNF-α inhibitors, anti-integrin agents, monoclonal antibodies, and non-biological small molecules. Despite the approval of numerous medications, most clinical trials have demonstrated a response rate of less than 60%. Primary and secondary non-response cases, along with both short- and long-term adverse events, remain prevalent (3). The interaction between gut microbiota and IBD has been extensively investigated. Recent studies have further validated that fecal microbiota transplantation (FMT) yields significant improvements in alleviating IBD symptoms (4). Additionally, interventions involving probiotics, prebiotics, postbiotics, and synbiotics present a distinct advantage over certain traditional treatment methods, as they do not induce drug resistance side effects, thereby offering a safer alternative for IBD management (5).

Currently, the etiology and pathogenesis of IBD remain incompletely understood. It is generally believed that IBD arises from a combination of factors, including genetic predisposition, environmental influences, disturbances in gut microbiota, and imbalances in the immune system (6). Among these, the disruption of gut microbiota homeostasis is considered a critical factor in both the initiation and maintenance of intestinal inflammation. The gut microbiota plays a crucial role in maintaining intestinal homeostasis, and dysbiosis can lead to intestinal inflammation (7). Numerous studies have demonstrated that the composition of gut microbiota in patients with IBD is significantly distinct from that of healthy individuals. IBD patients generally exhibit decreased bacterial diversity, specifically a reduction in *Firmicutes* and an increase in *Proteobacteria* (8). The decrease in the microbial diversity of IBD patients is due to the loss of commensal anaerobes, such as *Bacteroides*, *Eubacterium*, and *Lactobacillus* (9). UC patients had significantly lower abundances of the genera, *Prevotella*, *Eubacterium*, *Neisseria*, *Leptotrichia*, *Bilophila*, *Desulfovibrio*, and *Butyricimonas* in the inflamed sites compared to their respective sites in non-IBD controls (10). Several beneficial short-chain fatty acids (SCFAs) producing taxa, including *Faecalibacterium*, *Eubacterium*, and *Roseburia*, are enriched in patients who respond positively to FMT. In contrast, *Escherichia coli*, which belongs to the phylum *Proteobacteria*, is decreased in these patients (4). Compared to healthy controls, the abundance of *Bacteroides*, *Eubacterium*, *Faecalibacterium*, and *Ruminococcus* was significantly reduced at the genus level in fecal samples from patients with CD. The abundance of butyrate-producing bacterial species was reduced in CD patients compared to healthy individuals (11). Moreover, the metabolite profile of patients with IBD undergoes significant changes. Microbial-derived metabolites, including bile acids, SCFAs, and tryptophan metabolites, have been implicated in the pathogenesis of IBD (12). Given the central role of gut microbiota and its metabolites in the pathogenesis of IBD, probiotic interventions have been extensively studied as a potential strategy to modulate the gut microbiome and enhance gastrointestinal health.

Probiotics are a class of live microorganisms that confer benefits to the host by regulating the structure of intestinal flora, thereby exerting anti-inflammatory and other physiological effects (13, 14). *Phocaeicola vulgatus* has been shown to alleviate experimental mouse colitis by modulating the gut microbiota and immune response (15). *Alistipes shahii* has been reported to improve experimental colitis in mice by reducing intestinal epithelial damage and cytokine secretion (16). *Weissella confusa* alleviates experimental colitis in mice by downregulating inflammatory pathways and regulating gut microbiota (17). Numerous studies have demonstrated that the metabolites produced by probiotics can also mitigate IBD. *Enterobacter ludwigii* has been found to protect against dextran sodium sulfate (DSS)-induced colitis through choline-mediated immune tolerance (18). *Lactobacillus*-derived indole-3-lactic acid (ILA) has been proven to ameliorate colitis in mice born by cesarean section (19). Indole-3-acetic acid (IAA) has been shown to enhance the synthesis of R-equol, produced by *Bifidobacterium pseudolongum*, thereby mitigating the effects of DSS-induced colitis (20). Collectively, these studies indicate that probiotics and probiotics-derived metabolites possess significant potential for the prevention of IBD, further emphasizing the prospective value of probiotics in both the prevention and treatment of IBD.

*Eubacterium limosum*, a Gram-positive, obligately anaerobic, and rod bacterium, plays a crucial role in maintaining intestinal homeostasis and promoting host health (21). *E. limosum* is one of the predominant species in the human gut microbiota and is associated with host health. It has been identified as a potentially beneficial microorganism (22). *E. limosum* has been proven to produce butyrate, which accelerates intestinal epithelial growth and inhibits IL-6 production (23). *E. limosum* has been shown to ameliorate experimental colonic inflammation. Its metabolite, butyrate, enhances mucosal integrity through TLR4 signaling (24). These studies illustrate the potential of *E. limosum* in preventing IBD. However, the specific mechanisms and the detailed metabolic processes in the gastrointestinal tract remain poorly understood. Therefore, elucidation of the specific mechanisms of *E. limosum* in modulating intestinal inflammation may contribute to the treatment of IBD.

The *E. limosum* strain El1405 isolated from our laboratory had been demonstrated to possess anti-inflammatory effects, and it could inhibit colorectal cancer by reducing the levels of inflammatory factors within tumors (25). Given that patients with IBD exhibit a heightened risk of developing colorectal cancer, we hypothesize that this strain may also alleviate IBD. Consequently, this study investigated the protective effects of El1405 on DSS-induced colitis in mice. The results indicated that El1405 modulated gut microbiota, reduced levels of inflammatory cytokines, and produced anti-inflammatory metabolites, such as IAA and ILA, thereby effectively alleviating DSS-induced colitis. This study provides new insights into the anti-inflammatory properties of *E. limosum* and an experimental basis for the prevention and treatment of IBD, demonstrating that *E. limosum* may serve as a promising adjunctive treatment for IBD in the future.

## 2 Materials and methods

### 2.1 Bacterial culture

El1405 was recovered from healthy human feces collected and was verified by 16S rRNA gene sequencing and phylogenetic and phenotypic analyses (25). The culture method for El1405 is based on previous studies (25). The preservation number of El1405 was CGMCC NO. 31231.

### 2.2 Animal experiments

Specific-pathogen-free (SPF) female C57BL/6J mice, aged 5 to 6 weeks and weighing between 16 and 18 grams, were obtained from Vital River Lab Animal Technology Co., Ltd. in Beijing, China. The mice were housed in an environment maintained at a temperature of 23 ± 2 °C, with relative humidity set at 55% ± 5%, and subjected to a 12-hour light-dark cycle, all under specific pathogen-free conditions. After a one-week acclimatization period, the mice were randomly divided into three groups (8 mice per group): a control group (NC group), a phosphate-buffered saline (PBS)-treated colitis group (dextran sulfate sodium (DSS) group), and an *E. limosum* El1405-treated colitis group (El1405 group). Mice were orally administered 0.2 mL of PBS or 0.2 mL of El1405 (1×10^8^ CFU/0.2 mL per mouse, bacteria resuspended in PBS) for 14 days (day -7 - day -1). The NC group received an equal volume of PBS during the same period. After seven days, both the DSS and El1405 groups were administered a 3% solution of DSS (S0798, MP Biomedicals) for a consecutive duration of seven days to induce colitis (from day 0 to day 6), while the control group did not receive any DSS treatment. Specifically, the 3% DSS (freshly prepared) was incorporated into the drinking water for daily *ad libitum* consumption. The body weight of the mice was monitored daily throughout the study period. On day 8, the mice were sacrificed. Euthanasia was performed using a graded CO_2_ inhalation system (30% chamber displacement rate), followed by neck dislocation to ensure death. All procedures adhered strictly to the 2020 AVMA guidelines for animal euthanasia. The serum samples were collected and stored at -80 °C for the analysis of cytokines and metabolomics. The colon length was measured from the ileocecal junction to the anus, recorded, and photographed. The distal colons were harvested for histopathological examination, and cecal contents were collected for microbiota analysis.

All animal studies were approved by the Ethics Review Committee of the National Institute for Communicable Disease Control and Prevention at the Chinese Center for Disease Control and Prevention (Approval code: 2023–032).

### 2.3 Disease activity index (DAI) score

The DAI is a widely utilized measure for evaluating the severity of IBD. DAI was regularly assessed during the administration of DSS activity by scoring three distinct clinical parameters: stool consistency, weight loss, and hematochezia. The DAI scores were calculated as described in a previous study (18, 26).

### 2.4 Histological analysis

The distal colon tissue specimens were initially embedded in 4% paraformaldehyde and subsequently cross-sectioned perpendicular to the long axis of the colon for further pathological studies. Stained with hematoxylin-eosin (HE) and analyzed by histopathologists. The histological score was determined based on the criteria outlined in previous studies (27, 28). The total histological score for each mouse was calculated for comparison among the three groups. The tissue slices were scanned and imaged using the Pannoromic (3DHISTECH) panoramic section scanner. To achieve imaging at a magnification of 200-fold, the intestinal tissue area was selected using CaseViewer 2.4 (3DHISTECH) scanning software. Subsequently, five crypts were randomly chosen from each tissue section. For each selected crypt, we first measured its depth using Image-Pro Plus 6.0 (Media Cybernetics) analysis software and then counted the number of goblet cells it contained. The number of goblet cells per unit length (number/mm) was calculated using the formula: number of goblet cells per unit length = number of goblet cells/length of intestinal crypt.

Immunohistochemical assays were performed on the aforementioned tissue sections. The primary antibodies were incubated with mucoprotein 2 (MUC2), while the secondary antibodies were labeled with horseradish peroxidase specific to their respective species. The Aipathwell digital pathology image analysis software was utilized to automatically assess protein positivity, which was quantified using the histochemistry score (H-Score), which was determined based on criteria established in previous studies (29, 30). The H-Score quantifies the ratio of the positive area and staining intensity in each slice, converting these metrics into corresponding values. This approach facilitates a comprehensive semi-quantitative analysis of both the depth and degree of positive tissue immunostaining. The H-score was calculated using the formula H-score = ∑ (pi × i), where i represents the staining intensity grade score and pi denotes the percentage of cells positive for the corresponding grade. The staining intensity of positive cells is categorized into four grades: negative (no coloring, 0 points), weak positive (yellowish, 1), moderately positive (brownish-yellow, 2), and strong positive (tan, 3).

### 2.5 The enzyme-linked immunosorbent assay (ELISA)

Cytokine levels in the serum and colon, including TNF-α, IL-6, IL-17, IL-1β, TGF-β, LPS, IL-21, IL-22, and Granulocyte-Macrophage Colony-Stimulating Factor (GM-CSF) were evaluated by ELISA kits (Dogesce Beijing, China). Serum was diluted twofold and then tested by ELISA. For the colon, 0.1 g of colon tissue was weighed, and then 1 ml of PBS was added for grinding. Subsequently, ELISA detection was performed.

### 2.6 Cell culture

RAW264.7 cells were cultured in Dulbecco’s Modified Eagle Medium (DMEM, Gibco, USA) supplemented with 10% FBS (Sijiqing, China) at 37 °C and 5% CO_2_. The cell concentration was adjusted to 5×10^5^ cells/mL. Then, 100 μL of cells were seeded into each well of 96-well plates and cultured for 12 hours. The 0.01mg/mL IAA and ILA were added to RAW 264.7 cells, respectively, while 1 μg/mL lipopolysaccharide (LPS) was added to the cells, and co-cultured for 6 (TNF-α) or 20 (IL-1β and IL-6) hours. Cells with no treatment were used as a negative control, while cells treated with only LPS were used as a positive control. ELISA kit (Dogesce Beijing, China) was used to determine IL-1β, TNF-α, and IL-6 levels in cell supernatants.

### 2.7 16S rRNA gene sequencing

Cecal content samples from each mouse were collected on day 16, snap-frozen in liquid nitrogen, and then stored at -80 °C. The total microbial genomic DNA of cecal contents (Tiangen, China) was extracted using a DNA isolation kit. After extraction, the 16S rRNA gene full-length primers were designed as follows: Forward primer 27F: AGRGTTTGATYNTGGCTCAG/Reverse primer 1492R: TASGGHTACCTTGTTASGACTT (31).

The PCR products were purified with AMpure PB beads, quantified using a Qubit@ 2.0 Fluorometer (Thermo Scientific), and quality-assessed with the Agilent 2100 Bioanalyzer system (Agilent, USA). The qualified PCR products were sequenced on a Sequel II sequencer (PacBio, USA) (32). After sequencing, the quality inspection was conducted on the formed sequencing library, and the obtained high-quality circular consensus sequence (CCS) was processed serially, including barcode identification. The generated optimized CCS was clustered at the 97% similarity level (USEARCH, version 10.0), and its species classification was determined based on the serial composition of the operational taxonomic unit (OTU) (33). This study used the BMKCloud platform (https://www.biocloud.net) for the bioinformatics analysis. The highly qualified OTU was used to calculate the Alpha diversity, which presented the species richness and diversity of the samples, the Shannon index and the ACE index. The beta diversity was estimated through Principal Coordinates Analysis (PCoA), which was measured by calculating the Unweighted-Unifrac distances. The screening criteria of the linear discriminant analysis effect size (LEfSe) analysis was over 4. The one-way ANOVA statistical method was used to compare the differential abundance analysis of bacterial species. The 16S rRNA gene sequencing data from this study have been deposited in the NCBI Sequence Read Archive (SRA) database (Bioproject No: PRJNA1261401).

### 2.8 RNA sequencing and data analysis

According to the manufacturer's instructions, the colon RNA was extracted using TRIzol Reagent (Life Technologies, USA). The concentration and purity of the RNA were measured by NanoDrop 2000 (Thermo Fisher Scientific, USA). The RNA integrity was assessed by Bioanalyzer 2100 (Agilent Technologies, USA). Qualified RNA samples were utilized to construct cDNA libraries. The library construction process encompasses several steps, including mRNA enrichment, cDNA synthesis, fragmentation, end repair, adapter ligation, and PCR amplification, followed by quality control. The PCR products were subsequently purified using the AMPure XP system, and the quality of the library was assessed using the Agilent Bioanalyzer 2100 system. Following the manufacturer’s instructions, the libraries were sequenced on the Illumina NovaSeq platform, producing 150 bp paired-end reads. Sequence quality was evaluated using FastQC (v0.11.9), with low-quality bases and adapter contamination removed through fastp (v0.23.4), filtering the raw data in Fastq format to get clean data. The effective data were compared to the reference genomic sequences, with sequences that are either exactly matched or contain a single mismatch undergoing further analysis and annotation. The Hisat2 tool (v2.0.4) is employed for alignment with the reference genome (34). StringTie (v2.2.1), which utilizes the reference annotation-based transcript (RABT) assembly method, was used to identify known transcripts and predict new transcripts based on the results from the Hisat2 alignment (35). Differential expression analysis was conducted between the two groups using DESeq2 (36). Genes with a corrected *p value* < 0.05 and a fold change ≥ 1.5, as analyzed by DESeq2, were designated as differentially expressed (37). Subsequent analyses and data mining were performed on BMKCloud (www.biocloud.net). The heatmap of differentially expressed genes was generated using Morpheus (https://software.broadinstitute.org/morpheus). The raw data were deposited in the NCBI SRA database (Bioproject number: PRJNA1202937).

### 2.9 Quantitative real-time PCR

Total RNA was extracted from colon tissues using TRIzol Reagent and reverse-transcribed into cDNA with the PrimeScript™ RT Reagent Kit (TaKaRa, Japan) following the manufacturer’s protocol. The primers were synthesized by Tsingke Biotechnology (Beijing). Quantitative reverse transcription PCR (qRT-PCR) was conducted using the SYBR Green Realtime PCR Master Mix (Toyobo, Japan). *Gapdh* served as the internal reference, using the fold gene change = 2^−ΔΔCT^ method to determine the expression levels of the relevant genes. The primer sequences are provided in **Supplementary Table S1**.

### 2.10 Metabolomic analysis

A solution of methanol, acetonitrile, and water (2:2:1, v/v) was added to 100 μL of serum. The mixture was vortexed and subjected to low-temperature ultrasound for 30 minutes. The mixture was first incubated at -20 °C for 10 minutes, after which it was centrifuged at 14,000 g for 20 minutes at 4 °C. The supernatant was collected and processed via vacuum drying. For subsequent mass spectrometry analysis, 100 μL of an acetonitrile-water solution (1:1, v/v) was added to re-dissolve the dried sample; this was followed by vortex mixing and a second centrifugation step at 14,000 g for 15 minutes at 4 °C. Finally, the supernatant from this centrifugation was used for sample analysis.

Samples were analyzed using an Agilent 1290 Infinity LC ultra-high performance liquid chromatography (UHPLC) system (Agilent Technologies), which was equipped with both HILIC and C18 columns. For mass spectrometric analysis, an AB 6500+ QTRAP mass spectrometer (AB SCIEX) was employed. Multiple reaction monitoring (MRM) data acquisition and processing were performed using Agilent Mass Hunter Workstation Software (Version B.08.00, Agilent Technologies), with the original MRM data serving as the foundation for calculating metabolite content. All identified metabolites were classified and subjected to statistical analysis based on their chemical taxonomy. Subsequently, principal component analysis (PCA) was utilized to investigate the overall distribution patterns across different groups. Metabolites that met the criteria of fold change > 1 and *p value* < 0.05 were designated as differential metabolites, which were then used to generate a heatmap (18).

### 2.11 Statistical analysis

All experimental results were performed using GraphPad Prism 9.0 software. Data are expressed as the mean ± Standard Error of the Mean (SEM). Differences among multiple groups were assessed using one-way ANOVA followed by Dunnett’s multiple comparison test. The correlation between inflammatory factor concentrations and microbiota abundance was analyzed using Spearman’s rank correlation test. *p value* < 0.05 was considered statistically significant. NS means no significance. *p < 0.05, **p < 0.01, ***p < 0.001, and ****p < 0.0001.

## 3 Results

### 3.1 *E. limosum* ameliorated DSS-induced colitis in mice

**Figure 1:**
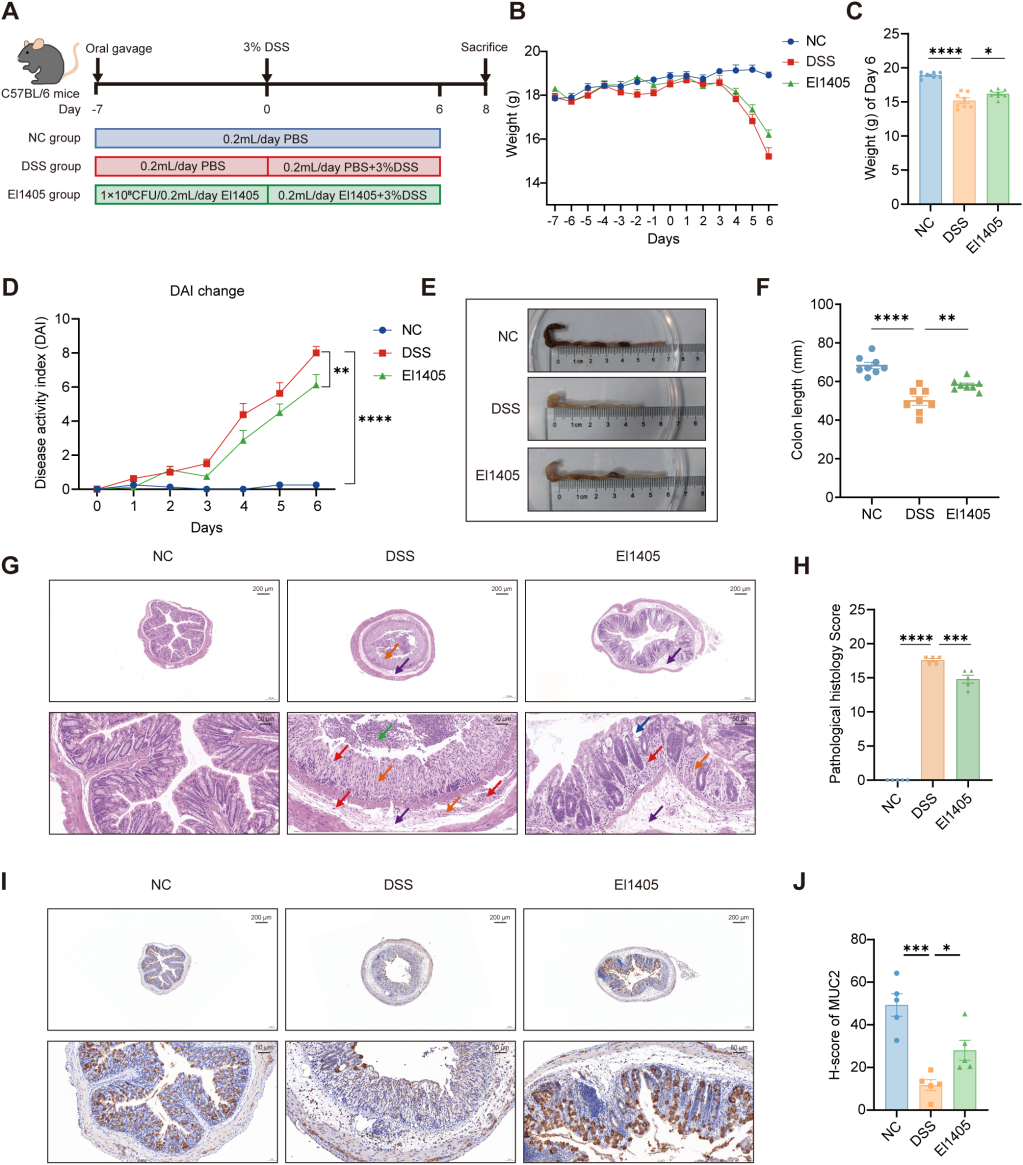
The effects of *E*. *limosum* on DSS-induced colitis in C57BL/6J mice. **(A)** Schematic of the experimental procedure (number of mice per group (n) =8); **(B)** Body weight change curves (n=8); **(C)** Weight on Day 6 (n=8); **(D)** DAI score of mice in the three groups from day 0 to 6 (n=8); **(E)** Representative images of the colons of mice from different treatment groups; **(F)** The colon length of mice (n=8); **(G)** Representative images of colon tissue sections stained with HE. Scale bar = 50 and 200 μm. In the NC group, a regular arrangement of mucosal epithelial cells was observed, and no abnormalities were noted in the intestinal gland tissue. In the DSS group, extensive ulcers were evident in the colon tissue, accompanied by a significant presence of lymphocytes, granulocytes, and necrotic cell fragments in the intestinal lumen. The mucosal epithelium and intestinal gland structures were severely compromised, with a notable absence of goblet cells and extensive proliferation of connective tissue, alongside widespread infiltration of lymphocytes and granulocytes. Additionally, there was extensive edema in the submucosal layer, characterized by massive connective tissue proliferation and a high degree of lymphocyte and granulocyte infiltration. In the El1405 group, partial ulceration of the colon tissue was observed, alongside a reduction in the number of intestinal glands and goblet cells, as well as significant proliferation of connective tissue. This condition was accompanied by scattered infiltration of lymphocytes and granulocytes, along with submucosal edema characterized by a loose arrangement of connective tissue and minimal infiltration of lymphocytes and granulocytes. The red arrows indicate lymphocyte infiltration, the blue arrows denote crypt dilation, the orange arrows highlight the disappearance of goblet cells and proliferation of connective tissue, the purple arrows illustrate diffuse edema in the submucosal layer, and the green arrows indicate the presence of granulocytes and necrotic cell fragments; **(H)** The pathological histology score of the colon (n=5); **(I)** Representative images of colon tissue sections stained for MUC2. Scale bar = 50 and 200 μm; **(J)** The histology score of the colon stained for MUC2 (n=5). Statistical comparison was performed by a one-way ANOVA followed by Dunnett’s multiple comparisons test. NC, normal control mice; DSS, DSS-induced mice treated with PBS; El1405, DSS-induced mice treated with El1405. Statistical significance is indicated as *p < 0.05, **p < 0.01, ***p < 0.001, and ****p < 0.0001.

To evaluate the beneficial effect of *E. limosum* on IBD, we employed a mouse model of DSS-induced colitis. Female C57BL/6J mice were pretreated with 1×10^8^ CFU *E. limosum* El1405 or PBS for 7 days. Following this pretreatment, the mice were administered the same dose of El1405 or PBS, along with oral treatment with 3% DSS for another 7 days (**Figure 1A**). An increasing trend in body weight was observed in the NC group; however, a decrease in body weight was noted in all mice from the DSS and El1405 groups (**Figure 1B**). Compared to the DSS group, the El1405 group showed significantly less weight loss and reduced DAI scores on day 6 (*p* < 0.05, **Figures 1C, D**). Compared to the NC group, the DSS group had significantly reduced colon length, whereas the El1405 group had significantly less colonic shortening compared to the DSS group (*p* < 0.01, **Figures 1E, F**). HE staining and histological analysis were performed to evaluate colonic mucosa injury systematically. The DSS group exhibited a significantly higher pathological histology score compared to the NC group (*p* < 0.001, **Figure 1H**). Specifically, the DSS group exhibited significant inflammation and cellular infiltration, along with a notable loss of goblet cells and crypts, hyperplasia of connective tissue, and diffuse edema within the submucosa (**Figure 1G**). Conversely, the El1405-treated group presented with a lower pathological histology score, characterized by diminished inflammation and cellular infiltration compared to the DSS group (*p* < 0.001, **Figures 1G, H**). Moreover, the El1405 group had a significantly increased number of goblet cells and the length of colon crypt, in comparison to the DSS group (*p* < 0.05, **Supplementary Figures S1A, B**). Subsequently, the expression level of MUC2 in intestinal epithelial cells was assessed using MUC2 immunohistochemical staining to elucidate its localization and relative abundance within intestinal tissue. The DSS group exhibited a significantly lower H-score compared to the NC group. Conversely, the El1405-treated group presented with a higher H-score, characterized by an increased number of goblet cells compared to the DSS group (*p* < 0.05, **Figures 1I, J**). These findings indicate that El1405 effectively ameliorated DSS-induced colitis in mice.

### 3.2 *E. limosum* modified the gut microbiota composition

**Figure 2:**
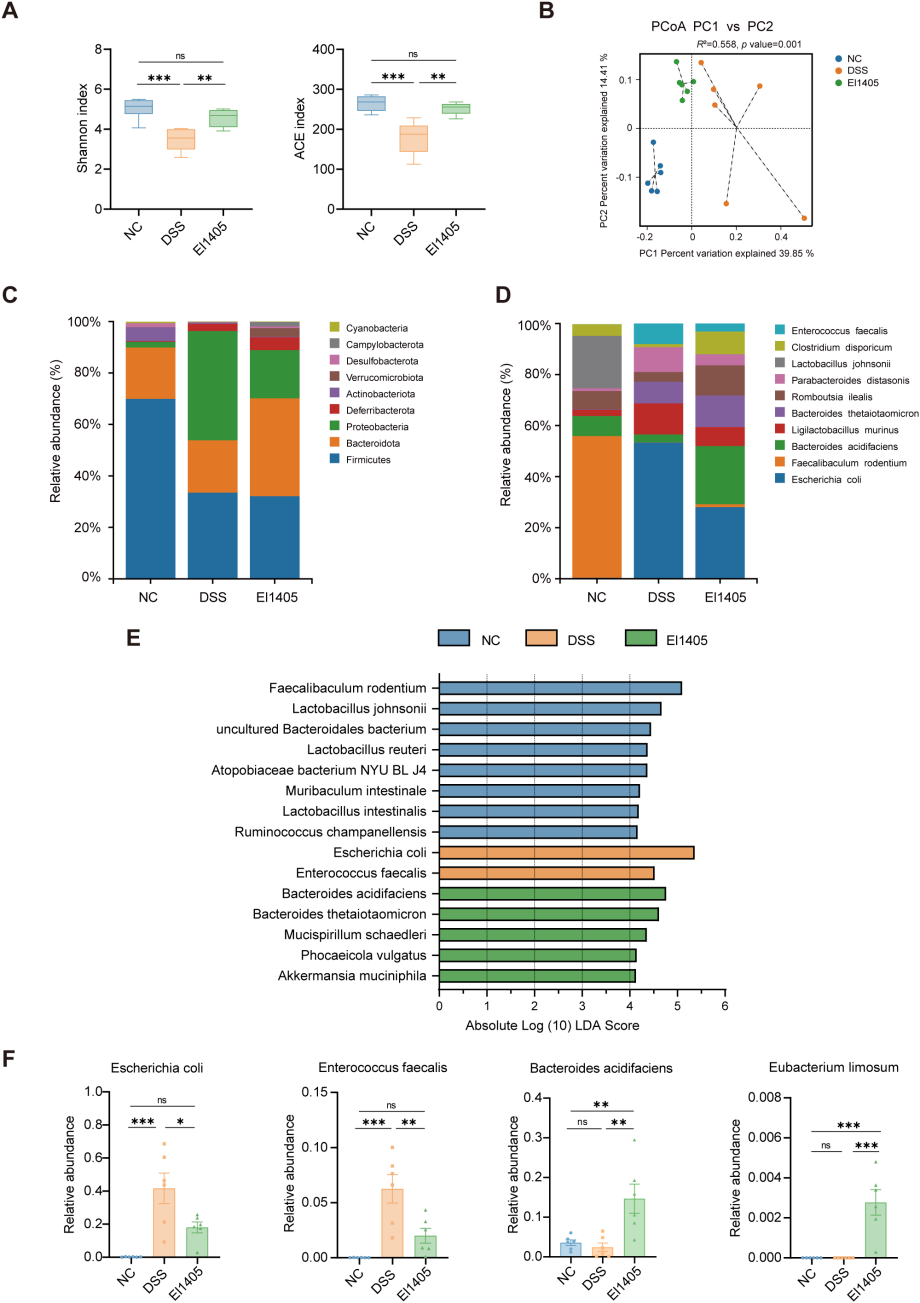
The effects of El1405 on the composition and abundance of intestinal microbiota in mice with DSS-induced colitis. **(A)** Boxplots of the Alpha diversity shown by the Shannon and ACE indices (n=6); **(B)** PCoA plots based on unweighted-unifrac distance (n=6); **(C, D)** Barplot analysis of microbiota composition profiling at the phylum and species level (top 10); **(E)** Overrepresented bacterial taxa among groups determined by LDA score with a threshold of 4; **(F)** Relative abundance of *E*. *coli*, *E*. *faecalis*, *B*. *acidifaciens*, and *E*. *limosum* (n=6). Statistical comparison was performed by a one-way ANOVA followed by Dunnett’s multiple comparisons test. Statistical significance is indicated as *p < 0.05, **p < 0.01, and ***p < 0.001.

To investigate the changes in microbiota composition, we conducted 16S rRNA gene sequencing on the cecal contents from the three groups of mice. The results indicated a significant reduction in gut microbiota diversity in the DSS group compared to the NC group, whereas the diversity in the El1405 treatment group was partially maintained (*p* < 0.05, **Figure 2A**, **Supplementary Figures S2A, B**). PCoA revealed that, based on the intestinal microbiota composition, the three groups of cecal contents samples were divided into three groups, and the samples from the El1405 group were more similar to those from the NC group (*p* < 0.05, **Figure 2B**). At the phylum level, *Proteobacteria* exhibited the highest relative abundance (42.46%) in the DSS group, *Firmicutes* was the predominant phylum (69.96%) in the NC group, and *Bacteroidota* was the predominant phylum (38.04%) in the El1405 group (**Figure 2C**). Compared with the NC group, the *Firmicutes/Bacteroidota* (F/B) ratio of the DSS group showed an increasing trend; compared with the DSS group, the F/B ratio of the El1405 group showed a decreasing trend. However, neither of these trends reached statistical significance (**Supplementary Figure S2C**). As shown in **Figure 2D**, the top three species with the highest relative abundance in the NC group were *Faecalibaculum rodentium*, *Lactobacillus johnsonii*, and *Bacteroides acidifaciens*. In the DSS group, the relative abundance of *F. rodentium* was reduced, while the *E. coli* group was the most dominant, accounting for 41.63%. The relative abundance of *B. acidifaciens* and *Bacteroides thetaiotaomicron* was increased in the El1405 group. LEfSe analysis showed that at the species level, *E. coli* and *Enterococcus faecalis* were significantly enriched in the DSS group, conversely, *B. acidifaciens, B. thetaiotaomicron, Mucispirillum schaedleri, Phocaeicola vulgatus*, and *Akkermansia muciniphila* were enriched in the El1405 group (**Figure 2E**). Furthermore, one-way ANOVA analysis confirmed that the relative abundance of *E. coli* and *E. faecalis* in the DSS group was significantly higher than that in the other two groups, in contrast, the relative abundance of *B. acidifaciens* and *E. limosum* in the El1405 group was higher than that observed in the other two groups (*p* < 0.05, **Figure 2F**). These results demonstrated that El1405 restored the altered gut microbiota composition in DSS-induced colitis mice.

### 3.3 *E. limosum* alleviates DSS-induced colitis in mice through downregulation of the IL-17 signaling pathway

**Figure 3:**
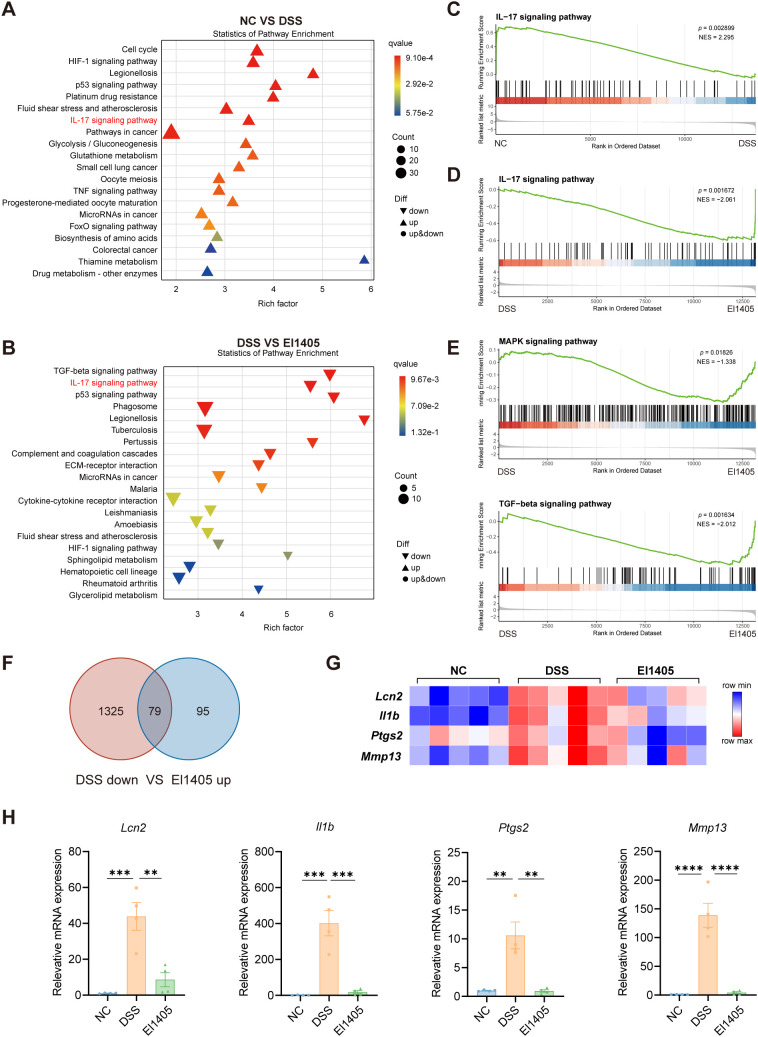
The effects of El1405 treatment on the transcriptomic profile of colon tissue in mice with DSS-induced colitis. **(A)** KEGG pathway enrichment analysis of the DEGs that were up-regulated in the DSS group compared to the NC group; **(B)** KEGG pathway enrichment analysis of the DEGs that were down-regulated in the El1405 group compared to the DSS group; **(C)** GSEA of the IL-17 signaling pathway gene set in the DSS group compared to the NC group; **(D)** GSEA of the IL-17 signaling pathway gene set in the El1405 group compared to the DSS group; **(E)** GSEA of the MAPK and TGF-β signaling pathway gene set in the El1405 group compared to the DSS group; **(F)** The Venn diagram illustrates the overlapping genes that were up-regulated in the DSS group and down-regulated in the El1405 group; **(G)** Heatmap of DEGs in the IL-17 signaling pathway; **(H)** The mRNA levels of *Lcn2*, *Il1β*, *Ptgs2*, and *Mmp13* in colon tissues. Statistical comparison was performed by a one-way ANOVA followed by Dunnett’s multiple comparisons test. Statistical significance is indicated as **p < 0.01, ***p < 0.001, and ****p < 0.0001.

RNA sequencing was conducted on colon tissues to elucidate the molecular mechanisms of El1405 in reducing colitis. Differentially expressed genes (DEGs) were screened in comparison to the respective control groups (*p <* 0.05, fold change > 1.5). Compared with the NC group, there were 757 up-regulated genes and 1404 down-regulated genes in the DSS group (**Supplementary Figure S3A**). KEGG enrichment analysis revealed that compared to the NC group, the up-regulated DEGs were significantly enriched in cell cycle, HIF, p53, IL-17, and TNF signaling pathways in the DSS group (**Figure 3A**). Compared with the DSS group, 174 genes were up-regulated and 290 genes were down-regulated in the El1405 group (**Supplementary Figure S3B**). Among the DEGs downregulated in the El1405 group, those in the TGF-β, IL-17, and p53 signaling pathways were significantly enriched (**Figure 3B**). In addition, gene set enrichment analysis (GSEA) showed that IL-17 and p53 signaling pathways were up-regulated (|NES| >1, *p <* 0.05) in the DSS group compared with the NC group (**Figure 3C**, **Supplementary Figure S4A**). In contrast, compared with the DSS group, IL-17, p53, MAPK, and TGF-β signaling pathways were down-regulated (|NES| >1, *p <* 0.05) in the 1405 group (**Figures 3D, E**, **Supplementary Figure S4B**). The Venn plot shows that 79 overlap genes were up-regulated in the DSS group and down-regulated in the El1405 group (**Figure 3F**). Next, we selected the genes related to the IL-17 pathway from these 79 DEGs and plotted them onto a heatmap (**Figure 3G**, **Supplementary Table S2**). The heatmap illustrated the four core DEGs in the IL-17 signaling pathway, including *Lcn2*, *Il1β*, *Ptgs2*, and *Mmp13*. Subsequently, qRT-PCR was used to verify these four core DEGs. It was noteworthy that the expression levels of these genes were significantly lower in the El1405 group compared to the DSS group, which aligned with the results of RNA sequencing (*p <* 0.05, **Figure 3H**). These results indicated that El1405 effectively inhibited the increase of the IL-17 signaling pathway in DSS-induced colitis in mice.

### 3.4 *E. limosum* administration down-regulated pro-inflammatory cytokines in DSS-induced colitis in mice

**Figure 4:**
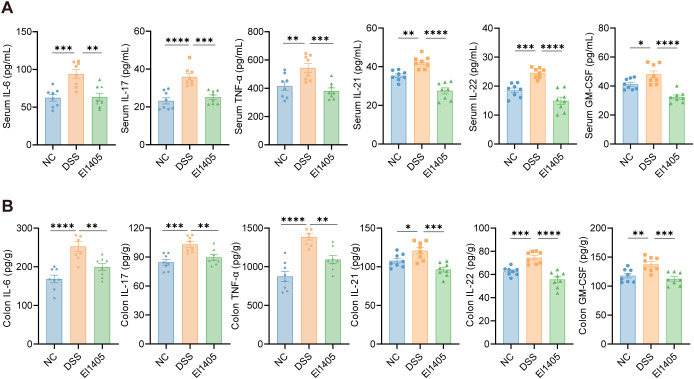
The effects of El1405 treatment on cytokines in DSS-induced mice. **(A)** Levels of cytokines in the serum of mice; **(B)** Levels of cytokines in the colon of mice. Number of mice per group = 8 for each group. Statistical comparison was performed by a one-way ANOVA followed by Dunnett’s multiple comparisons test. Statistical significance is indicated as *p < 0.05, **p < 0.01, ***p < 0.001, and ****p < 0.0001.

To further evaluate the effect of El1405 on the inflammatory response, we measured the levels of typical inflammatory cytokines (IL-6, IL-17, TNF-α, IL-21, IL-22, and GM-CSF) in the serum and colon of mice, which are associated with the IL-17 pathway based on RNA sequencing results. Specifically, IL-21, IL-22, and GM-CSF, which are cytokines secreted by Th17 cells and contribute to the Th17 cell-mediated inflammatory response. Compared to the NC group, the concentrations of IL-6, IL-17, TNF-α, IL-21, IL-22, and GM-CSF were significantly elevated in both the serum and colon of the mice following DSS treatment (*p* < 0.05). However, treatment with El1405 resulted in a significant decrease in these pro-inflammatory cytokines (*p* < 0.05, **Figures 4A, B**). Moreover, serum LPS and IL-1β levels were significantly higher in the DSS group than in the NC group (*p* < 0.01, **Supplementary Figures S5A, B**), while the El1405 group showed a significant reduction in these factors compared to the DSS group (*p* < 0.05). We also observed that TGF-β levels were lower in El1405-treated mice compared to DSS-treated mice, which was consistent with the RNA sequencing results indicating a decrease in TGF-β signaling pathway activity (*p* < 0.01, **Supplementary Figure S5C**). These findings suggest that El1405 inhibits the secretion of pro-inflammatory cytokines in both the serum and colon of the mice.

To explore the relationship between gut microbiota and immune factors, we analyzed the correlations between the relative abundance of significantly different species and serum/colon cytokine levels. Both *E. coli* and *E. faecalis* exhibited positive correlations with colon TNF-α, IL-6, and IL-17 levels, suggesting their potential role in promoting colitis development. In contrast, the microbiota enriched in the NC group, including *F. rodentium, L. johnsonii*, uncultured *Bacteroidales* bacterium, *Lactobacillus reuteri*, *Muribaculum intestinale*, *Lactobacillus intestinalis*, and *Ruminococcus champanellensis*, was negatively associated with TNF-α, IL-6, and IL-17 in the colon (*p* < 0.05, **Supplementary Figure S6**). These findings indicate that both *E. coli* and *E. faecalis* exhibited positive correlations with pro-inflammatory cytokines. This suggests that the El1405 intervention decreases the abundance of pathogenic bacteria associated with pro-inflammatory responses, thereby mitigating the effects of chronic colitis.

### 3.5 *E. limosum* alleviates DSS-induced colitis in mice through altering the metabolism of serum

**Figure 5:**
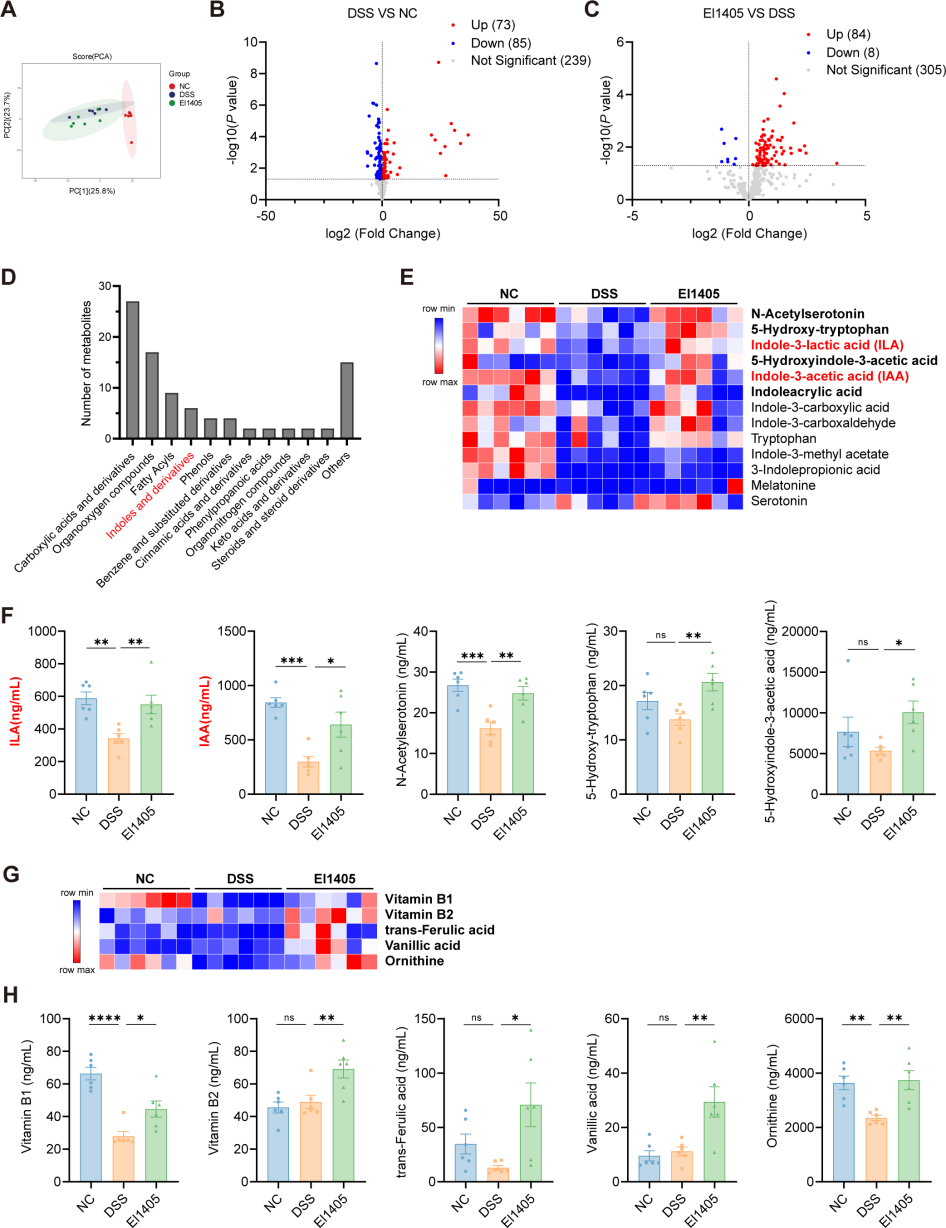
The effects of El1405 treatment on serum metabolome in DSS-induced mice. **(A)** PCA analysis of serum metabolome (number of mice per group (n) =6); **(B)** The volcano plot of serum differential metabolites in the DSS and NC group (n=6); **(C)** The volcano plot of serum differential metabolites in the El1405 and DSS group (n=6); **(D)** Histogram of metabolite classification; **(E)** Heatmap of indole derivatives from serum samples (n=6); **(F)** The levels of indole derivatives in serum (n=6); **(G)** Heatmap of beneficial metabolites from serum samples (n=6); **(H)** The levels of beneficial metabolites in serum (n=6). Statistical comparison was performed by a one-way ANOVA followed by Dunnett’s multiple comparisons test. Statistical significance is indicated as *p < 0.05, **p < 0.01, ***p < 0.001, and ****p < 0.0001.

Targeted metabolomics was employed to quantify the serum metabolic profiles of mice. A total of 397 metabolites were identified and classified (**Supplementary Table S3**). PCA revealed significant differences in serum metabolite profiles among these three groups (**Figure 5A**). A total of 73 metabolites were identified as significantly up-regulated, while 85 metabolites were found to be significantly down-regulated in the DSS group compared to the NC group (fold change > 1 and *p <* 0.05) (**Figure 5B**). Additionally, compared to the DSS group, the El1405 group demonstrated a significant increase in the levels of 84 metabolites, whereas the levels of 8 metabolites exhibited a significant decrease (fold change > 1 and *p <* 0.05) (**Figure 5C**). Analyzing the classification of 92 metabolites with significant differences in concentrations between the El1405 and DSS groups, we found that six of the differential metabolites were indole derivatives (**Figure 5D**). The heatmap illustrated the differences in serum indole derivatives between the DSS and El1405 groups of mice. Mice treated with El1405 exhibited significantly elevated serum levels of n-acetylserotonin, 5-hydroxy-tryptophan, ILA, 5-hydroxyindole-3-acetic acid (5-HIAA), and IAA when compared to those treated with DSS (*p <* 0.05, **Figures 5E, F**). Notably, IAA and ILA levels were significantly lower in the DSS group than the NC group, but higher in the El1405 group than the DSS group (**Figure 5E**, **Supplementary Tables S4**, **S5**). Based on the conclusion we previously drew from *in vitro* experiments regarding the production of ILA and IAA by El1405 (25), these results suggest that IAA and ILA may be the main metabolites of EI1405 in alleviating IBD. Furthermore, we found that IAA markedly inhibited the secretion of IL-1β, IL-6, and TNF-α in LPS-induced RAW264.7 cells (*p* < 0.05, **Supplementary Figure S7**). However, ILA showed no inhibitory effect on the secretion of these cytokines. We also observed that in the serum of mice treated with El1405, the contents of metabolites such as vitamin B1, vitamin B2, trans-ferulic acid, vanillic acid, and ornithine were significantly increased (*p <* 0.05, **Figures 5G, H**). Previous studies have reported that these metabolites were closely related to the alleviation of IBD (38–42).

## 4 Discussion

Previous studies have indicated that, compared with healthy individuals, the abundance of *Eubacterium* in the intestines of patients with IBD is significantly reduced, while bacterial groups such as *E. coli*, *B. fragilis*, and *Ruminococcus* are significantly increased (9, 11, 43). As an important beneficial bacterium in the intestine, *E. limosum* has been reported to have anti-inflammatory properties (24). In this study, we found that the *E. limosum* El1405 could effectively alleviate colitis in mice induced by DSS, specifically, by reducing weight loss, lowering DAI and histopathological score, inhibiting colonic shortening, and reducing the infiltration of inflammatory cells in the colonic tissue.

IBD is frequently associated with disturbances in intestinal flora. The intestinal microbiota composition of IBD patients differs significantly from that of healthy people, with a common characteristic among IBD patients being reduced bacterial diversity (7). Patients with IBD exhibit elevated levels of *Proteobacteria* and reduced abundance of *Bacteroides, Eubacterium*, and *Faecalibacterium* compared with healthy individuals (44). In this study, the El1405 intervention in DSS-induced colitis mice significantly altered the composition of the intestinal flora and increased bacterial diversity. It led to a notable increase in the abundance of *B. acidifaciens*, *B. thetaiotaomicron*, *M. schaedleri*, *P. vulgatus*, and *A. muciniphila* and a decrease in the abundance of pathogenic species, such as *E. coli* and *E. faecalis*. Numerous studies have linked these two species to IBD (15, 28, 45–47). *B. acidifaciens* could regulate the gut flora to improve DSS-induced colitis (45). *B. thetaiotaomicron* has been reported to decrease colon inflammation in colitis animal models (28). *M. schaedleri* has been reported to combat colitis by restricting *Salmonella* infection and inhibiting the expression of virulence factors (46). *P. vulgatus* alleviates colitis in experimental mice by regulating gut microbiota and immune response (15). *A. muciniphila*-derived extracellular vesicles have been shown to increase the abundance of *Firmicutes* while decreasing *Proteobacteria* in colitis mice, thereby modulating the intestinal barrier (47). On the other hand, adherent-invasive *E. coli* has been reported to enhance epithelial permeability, thereby promoting the development of IBD (48). *E. faecalis* has been reported to induce IBD, which even progresses to rectal dysplasia and adenocarcinoma in the IL-10 knockout mice (49). Thus, *E. limosum* El1405 might ameliorate IBD conditions by altering the composition of the intestinal microbiota. Specifically, it enhanced the abundance of beneficial bacteria in the gut against IBD while simultaneously reducing the presence of harmful bacteria.

One of the characteristics of IBD is the increased apoptosis of intestinal epithelial cells, which is closely related to elevated levels of TNF, inducible nitric oxide synthase, and p53 (50). Specifically, p53 mediates TNF-induced epithelial cell apoptosis in IBD (50). This study found that the p53 signaling pathway and TNF-α expression in the colon were down-regulated in the El1405 treatment group compared to the DSS group. Furthermore, in the El1405 group, the expressions of key genes related to the IL-17 signaling pathway (such as *Lcn2*, *Il-1β*, *Ptgs2*, and *Mmp13*) were significantly lower than those in the DSS group. The IL-17 signaling pathway can induce a cascade of pro-inflammatory factors, including TNF, IFN-γ, IL-22, lymphotoxin, IL-1β, and LPS, which are closely related to the pathogenesis of IBD (51). For example, *L. johnsonii* can alleviate DSS-induced colitis by down-regulating the IL-17 and TNF signaling pathways (52). Triptolide has been shown to alleviate colitis in IL-10-deficient mice by inhibiting the IL-6/STAT3 and IL-17 signaling pathways (53). Among the proteins related to the IL-17 pathway, LCN2 has been recognized as a fecal biomarker for patients diagnosed with UC (54). In mice, overexpression of LCN2 leads to severe colitis symptoms, while inhibiting LCN2 does not have this effect (55). IL-1β is expressed at higher levels in both plasma and colonic mucosal tissue of patients with IBD (56), significantly enhancing the pro-inflammatory response by recruiting and activating immune cells within the intestinal mucosa (57, 58), and participating in the disruption of the intestinal barrier and modulating the differentiation and function of helper T (Th) cells. Cyclooxygenase-2 (COX-2), encoded by the *Ptgs2* gene, is significantly involved in the inflammatory response induced by inflammatory factors such as IL-1β (59). While matrix metalloproteinases (MMPs), as members of the collagenase family, have a significantly elevated mRNA level in biopsy tissues of IBD patients (60), and activate TNF to disrupt the integrity of the intestinal epithelial barrier (61). These evidences all indicate that these genes are closely related to the heightened inflammatory response in IBD.

IL-6, IL-17, and TNF-α are pro-inflammatory cytokines closely related to the IL-17 pathway, playing crucial roles in inflammatory responses, immune regulation, and the pathology of various diseases (62, 63). IL-21, IL-22, and GM-CSF together constitute a Th17 cell-driven inflammatory regulatory network that plays a pivotal role in mediating the intestinal inflammatory response through the IL-17 pathway (64). IL-21 significantly enhances the activity of the IL-17 pathway by accelerating the differentiation and proliferation of Th17 cells (64, 65). Additionally, the overexpression of IL-22 can synergize with IL-17, further exacerbating intestinal inflammatory damage (66). GM-CSF recruits inflammatory cells such as monocytes and neutrophils, thereby promoting the progression of intestinal inflammation (67). In this study, we measured the expression levels of these six cytokines in serum and colon tissue. Our findings provide robust experimental evidence for the regulation of the molecular mechanisms underlying colitis alleviation through the IL-17 pathway by El1405. Further analysis revealed that the abundance of *E. coli* and *E. faecalis* was positively correlated with the levels of IL-6 and IL-17 in the serum, as well as TNF-α, IL-6, and IL-17 in the colon. These inflammatory factors showed more significant changes at the local colon level than in systemic (serum) levels. Previous studies have supported this correlation, for instance, adherent-invasive *E. coli* can activate intestinal Th17 cell subsets, promoting the production of pro-inflammatory factors, such as IL-17 and TNF-α, thereby exacerbating colitis (68). *E. faecalis* damages the intestinal barrier by producing lysophosphatidic acid and elevates IL-6 and IL-17 levels in blood (69). Moreover, both *E. faecalis* and *E. coli* can independently induce IL-17 secretion and cause colitis, while their coexistence leads to a more severe inflammatory response (70). Therefore, we speculate that the mechanism by which El1405 alleviates colitis may be by regulating the intestinal microbiota, significantly reducing the abundance of pathogens (especially *E. faecalis* and *E. coli)*, reducing the damage to the intestinal barrier, thereby reducing the local and systemic levels of IL-17, IL-6, and TNF-α, and ultimately alleviating colitis. However, the specific molecular mechanism of this regulatory process still needs to be further elucidated.

The metabolites produced by microbiota, especially tryptophan metabolites such as ILA and IAA, have been reported to play a significant role in ameliorating colitis. These indole derivatives typically exert their effects through their receptor, aryl hydrocarbon receptor (AhR) (71). Upon activation of the Trp-AhR pathway, the expression of downstream cytokines such as IL-22 and IL-17 is induced, which play a crucial role in regulating intestinal homeostasis (72). Clinical evidence shows that fecal ILA level is negatively correlated with IBD progression indicators, suggesting that ILA may maintain intestinal homeostasis by regulating epithelial-macrophage interactions (73). Additionally, ILA can ameliorate intestinal barrier damage and inhibit intestinal inflammation by activating the AhR-Nrf2 pathway and inhibiting the NF-κB pathway (74). IAA alleviates DSS-induced colitis in mice by altering the gut microbiome (20). ILA and IAA have been proven to mitigate intestinal inflammation and modulate the gut microbiota in DSS-induced colitis (75). This study found that El1405 could produce ILA and IAA in both *in vitro* and *in vivo* models, suggesting that these two metabolites might be the key anti-inflammatory factors for El1405 in improving IBD. Although it was currently unclear whether the sources of IAA and ILA in the intestine were solely from *E. limosum* or also from other microbiota (most likely contributed by both). In our previous study, the non-targeted metabolome analysis of fecal samples from El1405 intervention mice indicated that the concentrations of indole derivatives, such as IAA and ILA, in the feces were extremely low, failing to reach the effective detection threshold (25). If these metabolites can be detected under specific conditions, future studies can use germ-free mouse models to determine their exact sources. Moreover, the levels of vitamin B1, vitamin B2, transferulic acid, vanillic acid, and ornithine were significantly increased in the serum of El1405-treated mice, which have been reported to be associated with remission of IBD (38–42). Vitamin B1 has been reported to exhibit beneficial effects on chronic fatigue in IBD (38). Vitamin B2 has been shown to have protective effects on colitis in mice, reducing the production of anti-inflammatory factors (39). Vanillic acid has been shown to significantly inhibit the expression of COX-2 and the activation of transcriptional nuclear factor-κB p65 in colonic tissue, thereby reducing the severity of DSS-induced colitis (40). Trans-ferulic acid can be used in the synergistic treatment of IBD (41), and L-ornithine can enhance the effect of ustekinumab in the therapeutic effect of CD (42). Notably, although El1405 itself does not produce ornithine, the dominant bacterium *A. muciniphila*, which is significantly enriched in the intestine after its intervention, can promote the generation of ornithine (76). Therefore, we speculate that El1405 exerts its anti-inflammatory effects through two complementary mechanisms: directly producing anti-inflammatory metabolites and indirectly fostering a microbial community that generates beneficial metabolites, collectively contributing to the amelioration of colitis.

**Figure 6:**
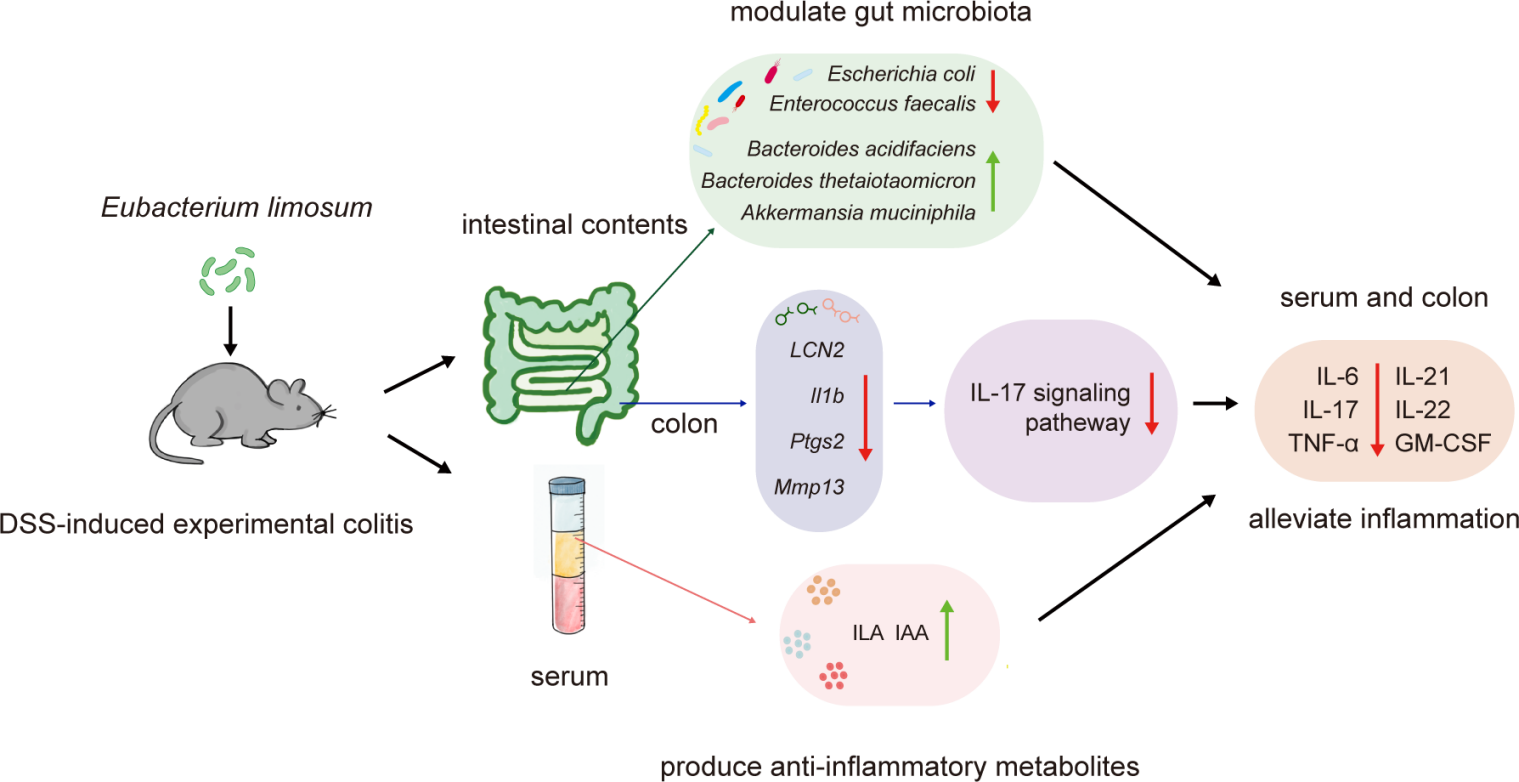
The schematic diagram summarises the anti-inflammatory effects of *Eubacterium limosum* El1405 on mice with DSS-induced colitis. *E. limosum* modulates the gut microbiota and produces anti-inflammatory metabolites, which downregulate IL-17 signaling, thereby alleviating the symptoms of DSS-induced colitis.

In conclusion, this study demonstrates the beneficial effects of the probiotic *E. limosum* El1405 on DSS-induced experimental colitis in mice. This effect is associated with the modulation of gut microbiota, a reduction in inflammatory cytokine levels, and the production of anti-inflammatory metabolites such as IAA and ILA, which collectively contribute to the downregulation of IL-17 signaling and the alleviation of DSS-induced colitis (**Figure 6**). However, the specific mechanisms involved warrant further investigation. Our study provides valuable insights into the potential use of *E. limosum* as a strategy for the prevention of IBD.

## 5 Limitations of this study

While this study confirms the protective effect of *E. limosum* El1405 on DSS-induced colitis, several key questions remain unresolved. It remains undetermined whether the gut microbiota is required for its anti-inflammatory action, or whether the increased anti-inflammatory metabolites in the bloodstream originate from *E. limosum* itself or other commensal bacteria. Furthermore, the precise mechanisms through which gut microbiota-derived metabolites, such as IAA and ILA, alleviate colitis require elucidation, including their specific anti-inflammatory pathways and therapeutic potential in IBD. A major limitation of this work is its focus on a UC-like model, leaving the efficacy of El1405 against CD. Thus, future studies employing germ-free models will help dissect the role of the microbiota, while mechanistic investigations are needed to clarify how bacterial metabolites mediate protection across different forms of IBD.

## Data Availability

The datasets presented in this study are available in online repositories. The raw data of 16S rRNA gene sequence data are publicly accessible in the NCBI Sequence Read Archive (SRA) database under Bioproject No: PRJNA1261401. The raw data for the RNA sequencing have been submitted to the NCBI SRA database (Bioproject number: PRJNA1202937).
