# Supplementary material for: Eubacterium limosum modulates gut microbiota and produces anti-inflammatory metabolites to alleviate DSS-induced colitis
Source: Front Immunol. 2025 Dec 16;16:1728808. doi: 10.3389/fimmu.2025.1728808 (PMC12747957; doi:10.3389/fimmu.2025.1728808)
Supplement: Supplementary file 1 [file Table1.docx]

Supplementary Material

**This file includes: Figures S1 to S6**


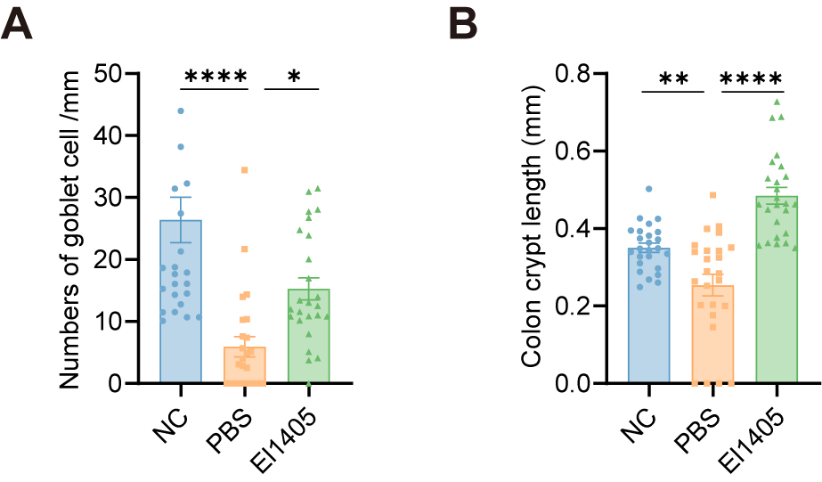


**Supplementary Figure S1.** The pathological histology of the colon. (A) Number of goblet cells in the colon per mm; (B) Colon crypt length. Statistical comparison was performed by a one-way ANOVA followed by Dunnett’s multiple comparisons test. Data are presented as mean ± Standard Error of Mean (SEM). ns, not significant; **p* < 0.05, ***p* < 0.01, ****p* < 0.001, and *****p* < 0.0001.


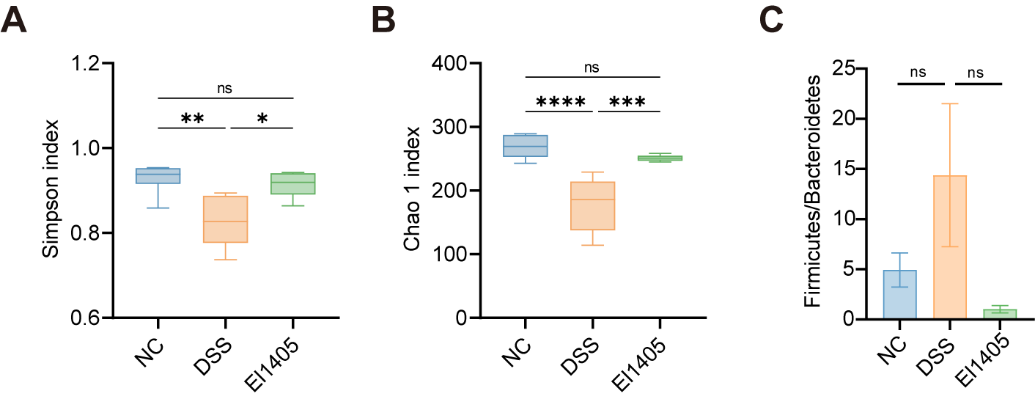


**Supplementary Figure S2.** Boxplots of the alpha diversity index among three groups, which were shown by the Simpson (A) and Chao 1 (B) indices (n=6); (C) The ratio of *Firmicutes*/*Bacteroidota*. Statistical comparison was performed by a one-way ANOVA followed by Dunnett’s multiple comparisons test. Data are presented as mean ± Standard Error of Mean (SEM). ns, not significant; **p* < 0.05, ***p* < 0.01, ****p* < 0.001, and *****p* < 0.0001.


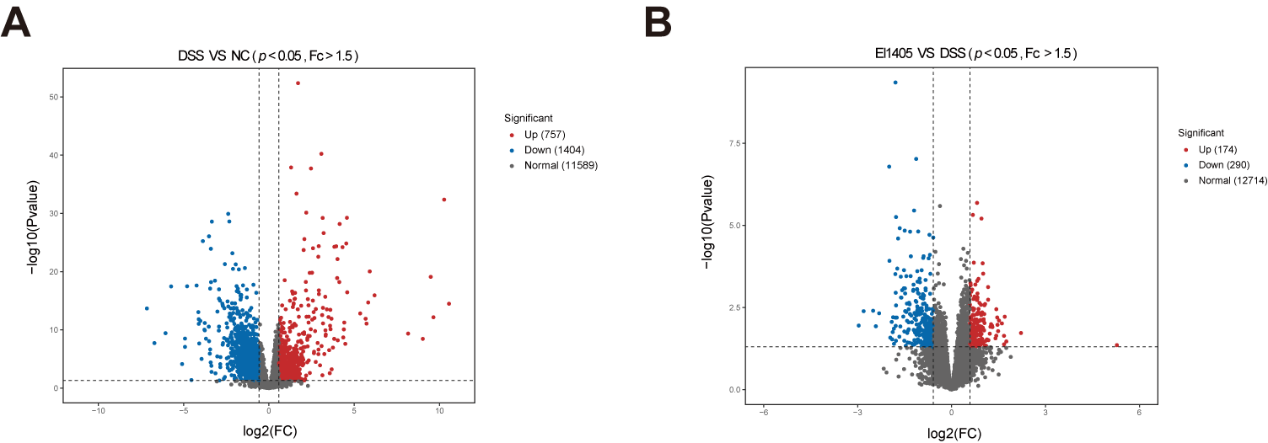


**Supplementary Figure S3.** Analysis of colon transcriptome. (A) The volcano plot of the DEGs in the DSS and NC group (n=5); (B) The volcano plot of the DEGs in the El1405 and DSS group (n=5).


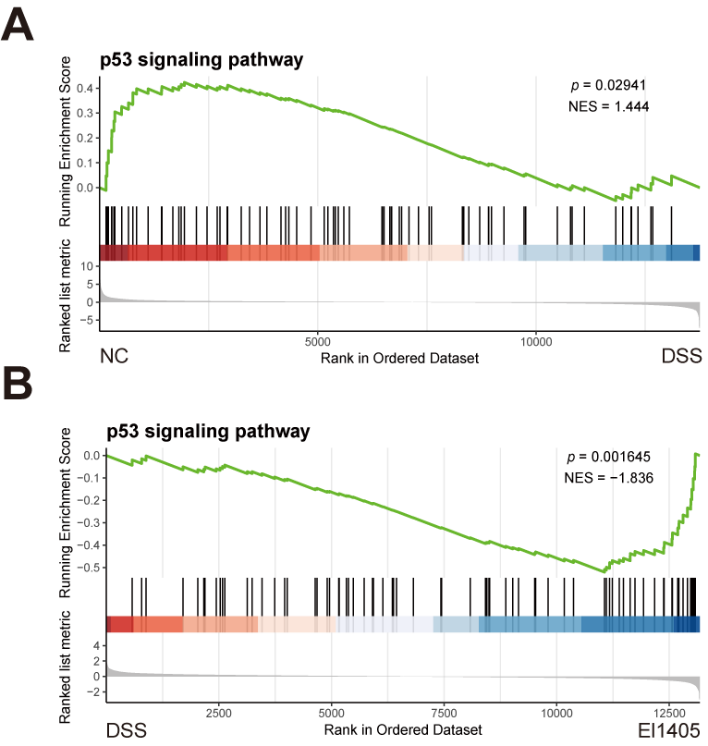


**Supplementary Figure S4.** Analysis of GSEA. (A) GSEA of the P53 signaling pathway gene set in the DSS group compared to the NC group (|NES| >1, P < 0.05); (B) GSEA of the P53 signaling pathway gene set in the El1405 group compared to the DSS group (|NES| >1, P < 0.05).


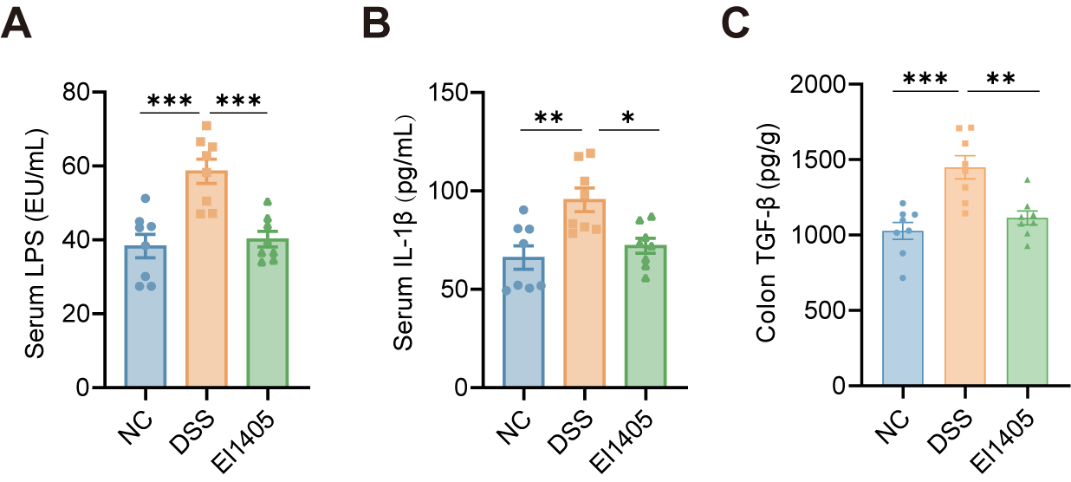


**Supplementary Figure S5.** The effects of El1405 treatment on cytokines in DSS-induced mice. (A) Levels of LPS in the serum of mice (n=8); (B) Levels of IL-1β in the serum of mice (n=8); (C) Levels of TGF-β in the colon of mice (n=8); Statistical comparison was performed by a one-way ANOVA followed by Dunnett’s multiple comparisons test. Data are presented as mean ± SEM. ns, not significant; **p* < 0.05, ***p* < 0.01, and ****p* < 0.001.


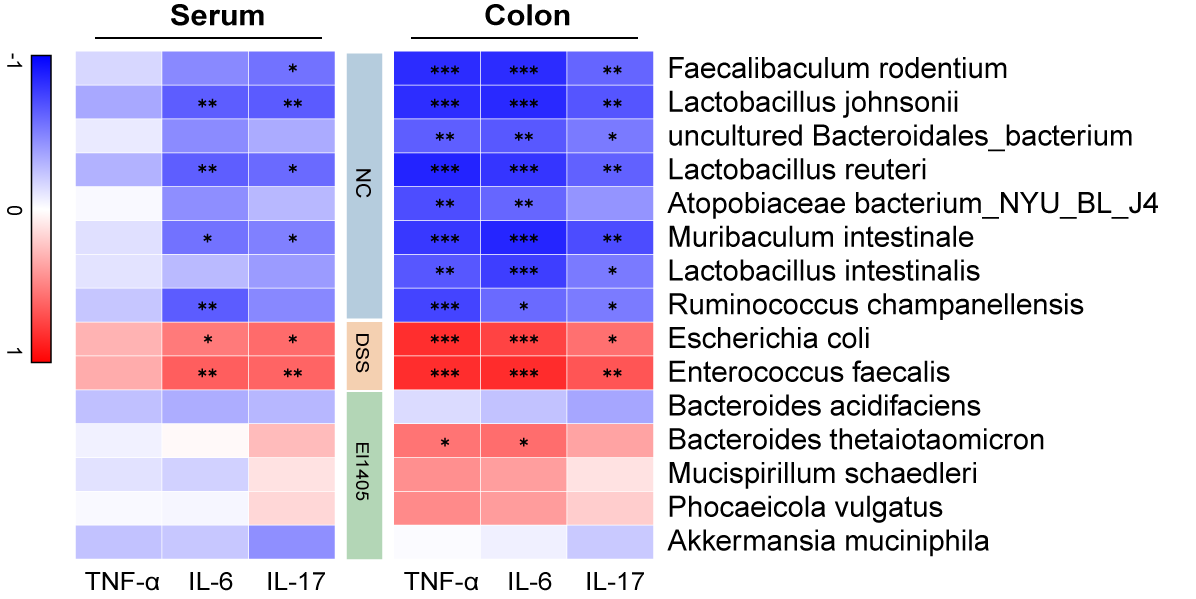


**Supplementary Figure S6.** Correlation analysis of species identified in LEfSe results with serum and colonic cytokines. Correlation analysis was conducted using Spearman correlation coefficients.


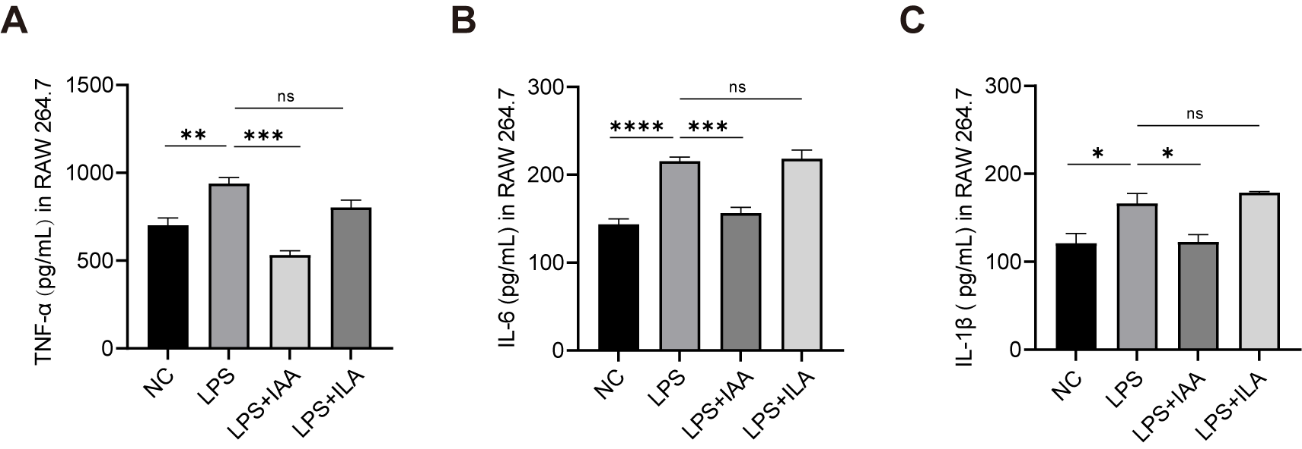


**Supplementary Figure S7.** The IAA and ILA significantly inhibited the secretion of TNF-α (A), IL-6 (B), and IL-1β (C) in LPS-induced RAW264.7 cells. Statistical comparison was performed by a one-way ANOVA followed by Dunnett’s multiple comparisons test. Data are presented as mean ± SEM. ns, not significant; **p* < 0.05, ***p* < 0.01, ****p* < 0.001, and *****p* < 0.0001.
